# Supplementary material for: Rural-urban disparities in full antenatal care utilization among women in Ethiopia: A further analysis of Mini-EDHS, 2019
Source: PLoS One. 2024 Nov 18;19(11):e0310901. doi: 10.1371/journal.pone.0310901 (PMC11573122; doi:10.1371/journal.pone.0310901)
Supplement: Attachment — Submitted filename: PONE-D-23-21294_reviewer.pdf [file pone.0310901.s004.pdf]

PLOS ONE

Rural-urban disparities in full antenatal care utilization among women in Ethiopia: a Multivariate decomposition analysis

--Manuscript Draft--

**Manuscript Number:**	PONE-D-23-21294
**Article Type:**	Research Article
**Full Title:**	Rural-urban disparities in full antenatal care utilization among women in Ethiopia: a Multivariate decomposition analysis
**Short Title:**	Rural-urban disparities in full antenatal care utilization among women in Ethiopia
**Corresponding Author:**	Elsabeth Addisu<br>Wollo University<br>Dessie, Dessie ETHIOPIA
**Keywords:**	Antenatal care; Women; multivariate decomposition; demographic and health survey; Ethiopia
**Abstract:**	Background: Even though full antenatal care service is a key intervention that creates the opportunity to provide all the necessary health services during pregnancy that aims to reduce maternal and new-born morbidity and mortality, there is still a gap in utilizing this service between rural and urban women. So, identifying the drivers of disparities will help to design an appropriate intervention.<br>Methods: A community-based cross-sectional study design was conducted. The data were collected from March 21, 2019 to June 28, 2019 in all regions of Ethiopia. Two stage cluster sampling techniques were used to select the study participants. This study has included about 3,927 (weighted samples) nationally representative samples of women aged from 15-49 years. A multivariate decomposition analysis technique was performed to see the rural-urban disparities in full antenatal care utilization explained by residence difference into components of endowments and coefficients.<br>Results: The prevalence of full antenatal care utilization was 43.25% (95% CI: 41.7%, 44.8%). The difference in the prevalence of full antenatal care utilization between rural and urban women was high (rural prevalence was 27.73%, while in urban areas it was 15.52%). Majority of the gap was explained by covariate distribution, which accounted for 76.84% and the rest 23.16% was due to the effect of covariate differences. Educational status, wealth, religion and region difference between urban and rural women explains most of the full antenatal care utilization disparities.<br>Conclusion and recommendations: There were significant full antenatal care utilization disparities among rural and urban women in Ethiopia. A larger portion of the rural-urban full antenatal care utilization gap was explained by differences in characteristics (endowment). So to narrow this gap, emphasis should be given to both resource distribution targeting to rural households, improvement of maternal education and creating a plat form to access information about the service and its relevance.
**Order of Authors:**	Elsabeth Addisu
	Niguss Cherie
	Tesfaye Birhane
	Zinet Abegaz
	Abel Endawekie
	Anissa Mohammed
	Dagnachew Melak
	Fekade Demeke Bayou
	Ahmed Hussien Asfaw
	Husniya Yasin
	Aregash Abebayehu Zerga

Powered by Editorial Manager® and ProduXion Manager® from Aries Systems Corporation

	Birhanu Wagaye
	Fanos Yeshanew Ayele
	Natnael Kebede
	Asnakew Molla Mekonen
	Mengistu Mera Mihiretu
	Amare Muche
	Yawkal Tsega
**Additional Information:**	
**Question**	**Response**
**Financial Disclosure**<br><br>Enter a financial disclosure statement that describes the sources of funding for the work included in this submission. Review the submission guidelines for detailed requirements. View published research articles from *PLOS ONE* for specific examples.<br><br>This statement is required for submission and **will appear in the published article** if the submission is accepted. Please make sure it is accurate.<br><br>**Unfunded studies**<br>Enter: *The author(s) received no specific funding for this work.*<br><br>**Funded studies**<br>Enter a statement with the following details:<br>• Initials of the authors who received each award<br>• Grant numbers awarded to each author<br>• The full name of each funder<br>• URL of each funder website<br>• Did the sponsors or funders play any role in the study design, data collection and analysis, decision to publish, or preparation of the manuscript?<br>• **NO** - Include this sentence at the end of your statement: *The funders had no role in study design, data collection and analysis, decision to publish, or preparation of the manuscript.*<br>• **YES** - Specify the role(s) played.<br><br>* typeset	The authors received no specific funding for this work
**Competing Interests**	All the authors have declared that no competing interests exist

Use the instructions below to enter a competing interest statement for this submission. On behalf of all authors, disclose any competing interests that could be perceived to bias this work—acknowledging all financial support and any other relevant financial or non-financial competing interests.<br><br>This statement is required for submission and **will appear in the published article** if the submission is accepted. Please make sure it is accurate and that any funding sources listed in your Funding Information later in the submission form are also declared in your Financial Disclosure statement.<br><br>View published research articles from *PLOS ONE* for specific examples.<br><br>**NO authors have competing interests**<br><br>Enter: *The authors have declared that no competing interests exist.*<br><br>**Authors with competing interests**<br><br>Enter competing interest details beginning with this statement:<br><br>*I have read the journal's policy and the authors of this manuscript have the following competing interests: [insert competing interests here]*<br><br>* typeset	
**Ethics Statement**<br><br>Enter an ethics statement for this submission. This statement is required if the study involved:<br><br>• Human participants<br>• Human specimens or tissue<br>• Vertebrate animals or cephalopods<br>• Vertebrate embryos or tissues<br>• Field research	The study used publicly available data from the 2019 Ethiopian Mini Demographic and Health Survey. This survey was approved by Inner City Fund (ICF) international as well as the EPHI Institutional Review Board to ensure that a protocol is in compliance with the U.S Department of Health and Human Services regulations for the protection of human subjects. Written informed consent was obtained from study participants and/or their parents or legal guardian(s).

Powered by Editorial Manager® and ProduXion Manager® from Aries Systems Corporation

Write "N/A" if the submission does not require an ethics statement.

General guidance is provided below. Consult the submission guidelines for detailed instructions. **Make sure that all information entered here is included in the Methods section of the manuscript.**

Format for specific study types

Human Subject Research (involving human participants and/or tissue)

- Give the name of the institutional review board or ethics committee that approved the study
- Include the approval number and/or a statement indicating approval of this research
- Indicate the form of consent obtained (written/oral) or the reason that consent was not obtained (e.g. the data were analyzed anonymously)

Animal Research (involving vertebrate animals, embryos or tissues)

- Provide the name of the Institutional Animal Care and Use Committee (IACUC) or other relevant ethics board that reviewed the study protocol, and indicate whether they approved this research or granted a formal waiver of ethical approval
- Include an approval number if one was obtained
- If the study involved *non-human primates*, add *additional details* about animal welfare and steps taken to ameliorate suffering
- If anesthesia, euthanasia, or any kind of animal sacrifice is part of the study, include briefly which substances and/or methods were applied

Field Research

Include the following details if this study involves the collection of plant, animal, or other materials from a natural setting:

- Field permit number
- Name of the institution or relevant body that granted permission

Powered by Editorial Manager® and ProduXion Manager® from Aries Systems Corporation

**Data Availability**<br><br>Authors are required to make all data underlying the findings described fully available, without restriction, and from the time of publication. PLOS allows rare exceptions to address legal and ethical concerns. See the PLOS Data Policy and FAQ for detailed information.<br><br>A Data Availability Statement describing where the data can be found is required at submission. Your answers to this question constitute the Data Availability Statement and **will be published in the article**, if accepted.<br><br>**Important:** Stating 'data available on request from the author' is not sufficient. If your data are only available upon request, select 'No' for the first question and explain your exceptional situation in the text box.<br><br>Do the authors confirm that all data underlying the findings described in their manuscript are fully available without restriction?	Yes - all data are fully available without restriction
**Describe where the data may be found in full sentences. If you are copying our sample text, replace any instances of XXX with the appropriate details.**<br><br>• If the data are **held or will be held in a public repository**, include URLs, accession numbers or DOIs. If this information will only be available after acceptance, indicate this by ticking the box below. For example: *All XXX files are available from the XXX database (accession number(s) XXX, XXX.*).<br>• If the data are all contained **within the manuscript and/or Supporting Information files**, enter the following: *All relevant data are within the manuscript and its Supporting Information files.*<br>• If neither of these applies but you are able to provide **details of access elsewhere**, with or without limitations, please do so. For example:	The datasets analyzed in this study are available from the corresponding author upon reasonable request

Powered by Editorial Manager® and ProduXion Manager® from Aries Systems Corporation

Data cannot be shared publicly because of [XXX]. Data are available from the XXX Institutional Data Access / Ethics Committee (contact via XXX) for researchers who meet the criteria for access to confidential data.<br><br>*The data underlying the results presented in the study are available from (include the name of the third party and contact information or URL).*<br>• This text is appropriate if the data are owned by a third party and authors do not have permission to share the data.<br><br>* typeset	
Additional data availability information:	

Powered by Editorial Manager® and ProduXion Manager® from Aries Systems Corporation

Manuscript Click here to access/download;Manuscript;Submitted manuscript.docx

Rural-urban disparities in full antenatal care utilization among women in Ethiopia: a Multivariate decomposition analysis

Elsabeth Addisu*[1],Niguss Cherie[1], Tesfaye Birhane[1], Zinet Abegaz[1], Abel Endawekie[2], Anissa Mohammed[2], Dagnachew Melak[2] ,Fekade Demeke Bayou[2] , Ahmed Hussien Asfaw[3], Husniya Yasin[2] ,Aregash Abebayehu Zerga[3], Birhanu Wagaye[3], Fanos Yeshanew Ayele[3] , Natnael Kebede[4], Asnakew Molla Mekonen[5] , Mengistu Mera Mihiretu[5], Amare Muche[2], Yawkal Tsega[5]

Affiliations

[1]Department of Reproductive and Family Health, School of Public Health, College of Medicine and Health Sciences, Wollo University, Dessie, Ethiopia

[2] Department of Epidemiology and Biostatistics, School of Public Health, College of Medicine and Health Sciences, Wollo University, Dessie, Ethiopia

[3]Department of Public Health Nutrition, College of Medicine and Health Sciences, Wollo University, Dessie, Ethiopia

[4] Department of Health Promotion, School of Public Health, College of Medicine and Health Sciences, Wollo University, Dessie, Ethiopia

[5] Department of Health System and Management, School of Public Health, College of Medicine and Health Sciences, Wollo University, Dessie, Ethiopia

*Corresponding author: Elsabeth Addisu

E-mail address: elsabethko@gmail.com (EA)

Abstract

Background: Even though full antenatal care service is a key intervention that creates the opportunity to provide all the necessary health services during pregnancy that aims to reduce maternal and new-born morbidity and mortality, there is still a gap in utilizing this service between rural and urban women. So, identifying the drivers of disparities will help to design an appropriate intervention.

Methods: A community-based cross-sectional study design was conducted. The data were collected from March 21, 2019 to June 28, 2019 in all regions of Ethiopia. Two stage cluster sampling techniques were used to select the study participants. This study has included about 3,927 (weighted samples) nationally representative samples of women aged from 15-49 years. A multivariate decomposition analysis technique was performed to see the rural-urban disparities in full antenatal care utilization explained by residence difference into components of endowments and coefficients.

Results: The prevalence of full antenatal care utilization was 43.25% (95% CI: 41.7%, 44.8%). The difference in the prevalence of full antenatal care utilization between rural and urban women was high (rural prevalence was 27.73%, while in urban areas it was 15.52%). Majority of the gap was explained by covariate distribution, which accounted for 76.84% and the rest 23.16% was due to the effect of covariate differences. Educational status, wealth, religion and region difference between urban and rural women explains most of the full antenatal care utilization disparities.

Conclusion and recommendations: There were significant full antenatal care utilization disparities among rural and urban women in Ethiopia. A larger portion of the rural-urban full antenatal care utilization gap was explained by differences in characteristics (endowment). So

to narrow this gap, emphasis should be given to both resource distribution targeting to rural households, improvement of maternal education and creating a plat form to access information about the service and its relevance.

Keywords: Antenatal care, women, multivariate decomposition, demographic and health survey, Ethiopia

Introduction

Full antenatal care (ANC) is defined as 4 or more antenatal visits, at least 1 tetanus toxoid (TT) vaccination and consumption of iron folic acid (IFA) for a minimum of 100 days (1). The world health organization (WHO) aimed that every pregnant woman and new-born gets quality care during pregnancy, child birth and postnatal period for the world (2). ~~Nevertheless,~~ in 2015 nearly 303,000 women and adolescent girls dead due to pregnancy and child birth related complications (3). At the same time, of 99% of maternal deaths and 2.6 million still births which can be prevented were in low-resource settings (4). ANC reduces maternal and perinatal morbidity and mortality both directly, through detection and treatment of pregnancy-related complications, and indirectly, through the identification of women and girls at increased risk of developing complications during labour and delivery, thus ensuring referral to an appropriate level of care (5). Moreover, as indirect causes of maternal morbidity and mortality, such as HIV and malaria infections, contribute to approximately 25% of maternal deaths and near-misses(6).

A cohort study in Ethiopia, reported that having four or more ANC visits was significantly associated with 81.2%, 61.3%, 52.4% and 46.5% reduction in postpartum haemorrhage, early neonatal death, preterm labour and low-birth weight, respectively(7). A study from 27 selected countries in Sub- Saharan Africa showed that 12.2% difference between urban and

rural areas in the use of antenatal care service(8). According to EDHS 2016 report women having 4+ANC visit were 62.7% and 27.3% in urban and rural areas respectively(9).

Full antenatal care utilization has been associated with different factors. Those includes women's education(8, 10-15), mass media exposure(8, 10, 13), household wealth index(8, 10, 13-15) residence(11, 14), unintended pregnancy(11, 12) and perceived the right initiation time(11, 12), long distance from health facility(10, 16), living in city administration, community women literacy, and Pastoralist region(10).

However, the decomposition analysis method helps to decompose inequalities in full antenatal care utilization in urban-rural settings. Because though evidence showed a difference in service uptake it was not rigorously explained using the decomposition method yet. In order to design appropriate intervention strategies and programs which minimizes the gap between rural and urban areas, appreciating of the contributing factors of the rural-urban differences of full antenatal care utilization has a great importance. Hence, this study aimed to identify the source of variation in full antenatal care utilization between the rural and urban areas in Ethiopia by employing multivariate decomposition analysis technique.

Methods and Materials

Source of data and Populations

This study was based on the 2019 Mini-Ethiopian Demographic health survey (MEDHS) dataset. The nationally representative DHS survey collected information on several public health related topics comprising of five questionnaires which includes household, women's, anthropometry, and health facility and field workers. The data were obtained in STATA format from the website http://dhsprogram.com/data/.

The source population for this study was all women (15-49 years) who had a live birth in the five years preceding the survey residing in the selected clusters of 2019 Mini-Ethiopian Demographic and Health Survey. The sampled population was all reproductive-age women in each household in the enumeration area, and focused on their pregnancies during the 5 years preceding the survey.

Sample Size

This study included 3979 women aged 15-49years having live birth five years prior to the survey.

Sampling procedure

The 2019 MEDHS sample was stratified and selected in two stages. Each region was stratified into urban and rural areas, yielding 21 sampling strata. Samples of enumeration areas (EAs) were selected independently in each stratum in two stages. In the first stage, a total of 305 EAs (93 from urban and 212 from rural areas) were selected proportional to the size of the EA (based on the 2019 PHC frame) and with independent selection in each

sampling stratum. A household listing operation was carried out in all selected EAs from January to April, 2019. The resulting lists of households served as a sampling frame for the selection of households in the second stage. Some of the EAs selected for the 2019 MEDHS were large, with more than 300 households. To minimize the task of household listing, each large EA selected for the 2019 MEDHS was segmented. Only one segment was selected for the survey, with probability proportional to the segment size. Household listing was conducted only in the selected segment. In the second stage of selection, a fixed number of 30 households per cluster were selected with an equal probability through systematic selection from the newly created household listing. All women aged 15-49years who were either permanent residents or visitors and slept in the household the night before the survey were eligible for an interview(17).

Study variable Measurement

Dependent variable

Full antenatal care utilization was the outcome of interest and was assessed using self-reported data on pregnancy care of all live births that happened within 5 years of the dates of the surveys. For analysis, it was measured as a binary variable and was categorized as “1” if a woman had at least four ANC visit and less than 4 ANC visits as “0”.

Equity stratifier variable

Place of residence of the woman was the key grouping independent variable which has a binary outcome categorised into “urban” coded as 0 and “rural” coded as 1.

Independent variables

Socio-demographic characteristics: Women's age in years (15-24, 25-34 or 35-49), Women's education (no education, primary education, secondary education or higher education), Marital status (currently married or currently not married), religion (Orthodox, Muslim, protestant or others), wealth status (poor, middle or rich), region (pastoralist, urban, or agrarian), parity/number of living children (1 child, 2-3 children or 4+ children), and birth order (first, 2-3rd, 4-5th or 6+)

Operational definitions

Regions: Agrarian region(Amhara, Harari, Oromia, SNNP and Tigray) whose livelihood mainly based on agriculture considered and with better distribution of health facilities regions, pastoralist or emerging regions, whose livelihood based on mainly nomadism (Somali, Benishangul-Gumuz, Gambela and Afar) were with less access of healthcare services and urban regions, those livelihood based on employment and trade (Addis Ababa and Dire Dawa) (18, 19).

Wealth status: Based on the number and kinds of consumer goods they own, households are given scores. Principal component analysis was used to derive these scores. National wealth quintiles are compiled by assigning the household score to each usual household member, and then dividing the distribution into five equal categories, each comprising 20% of the population from the lowest poorest to richest for the current analysis we combine poorest and poorer as poor, richest and richer as rich and middle as it is (9).

Data processing and analysis

Data management and statistical analyses were conducted using STATA/MP 17.0 software. A secondary data were obtained from the 2019 MEDHS by sending a request letter to DHS program. The full data set in STATA file format were downloaded from MEASURE DHS website: https://dhsprogram.com after receiving authorization/permission letter to access the data set and important variables were extracted. After the data were cleaned and weighted considered descriptive statistics such as mean with standard deviation (SD), percentage, frequency tables and graphs were computed and presented to characterise the study populations. Survey design has been taken into account while the analysis was done. In order to identify determinants ~~of rural-urban differences in full ANC use among women in Ethiopia~~, the multivariate decomposition analysis technique was performed. The multivariate decomposition technique enabled us to see the determinants of the differences in an outcome (full ANC) for two groups (rural and urban). The differences in full ANC for rural and urban groups can be explained by differences in the level or distribution of the determinants of the outcome (explained component/covariates effect) and in the impacts of the determinants on the outcome (unexplained component/coefficients effect), and/or the interaction of the two components (20).

$$Y_{rural} - Y_{urban} = E + C = \sum_{k=1}^{k} W_{\Delta Xk} E + \sum_{k=1}^{k} W_{\Delta \beta k} C = \sum_{k=1}^{k} E_k + \sum_{k=1}^{k} C_k$$

$$E = \sum_{k=1}^{k} E_k = \sum_{k=1}^{k} W_{\Delta X_k} \{F(X_{rural}\beta_{rural}) - F(X_{urban}\beta_{rural}\}$$

$$C = \sum_{k=1}^{k} C_k = \sum_{k=1}^{k} W_{\Delta \beta k} \{F(X_{urban}\,\beta_{rural}) - F(X_{rural}\beta_{urban})\}$$

"E" refers to Endowment whereas; "C" refers to Coefficient

Powers et al. (21) developed a recent extension of the Oaxaca-Blinder method for non-linear outcome variables, the mvdcmp, which was used for the detailed decomposition. It was primarily intended for use in non-linear decomposition and convenient methods for dealing with path dependency (22) as well as overcoming the identification problem associated with the selection of the reference category when dummy variables are included as in the case of Oaxaca. Multivariate decomposition technique determines the high outcome group automatically and uses the low outcome group as a reference category (23).

Results

Socio-demographic and maternal characteristics

A total of 3927 (weighted samples) of women were included in this study. The mean age of maternal age with standard deviation was 28.9±0.1 years. Majority of 36.78% of rural and 13.95% of urban women were found in the age range of 25-34 years old. Regarding marital status more than two third 70.89% of rural and 24.33% urban women were currently married. Among respondents 26.61% of rural and 10.09% of urban residents were from Orthodox Christian religion. Nearly half (43.55%) of rural and (7.75%) of urban residents have no education. Regarding the wealth status distribution, almost 38.81% and 3.14% of rural and urban residents were poor in wealth status, respectively. More than three fourth (67.76%) of rural and 19.94% of urban residents were from an agrarian region. Almost half (36.01%) of the study participants from rural and 7.36% of urban areas have four and more live birth. Regarding birth order 20.66% of rural and 4.20% of urban area respondents were with a birth order of six and more child. (Table1)

Table 1: Characteristics of independent variables with full antenatal care and with stratifier variables of study participants using 2019 MEDHS, 2023; Weighted n=3927, unweighted n=3979

Variables	Variable Categories	Full antenatal care visit		Residence	
		Yes(1)	No(0)	Rural(1)	Urban(0)
Age in years	15-24	419(10.66%)	577(14.70%)	719(18.31%)	277(7.06%)
	25-34	926(23.59%)	1066(27.14%)	1444(36.78%)	548(13.95%)
	35-49	354(9.00%)	585(14.90%)	737(18.77%)	202(5.13%)
Marital status	Currently Married	1637(41.70%)	2102(53.53%)	2784(70.89%)	956(24.33%)
	Currently not married	61(1.55%)	127(3.22%)	116(2.97%)	71(1.81%)
Religion	Orthodox Christian	759(19.33%)	682(17.37%)	1045(26.61%)	396(10.09%)
	Muslim	505(12.86%)	835(21.26%)	1036(26.38%)	304(7.73%)
	Protestant	424(10.80%)	658(16.77%)	760(19.36%)	323(8.21%)
	Others*	11(0.27%)	53(1.35%)	59(1.51%)	4(0.11%)
Women education	No education	657(16.74%)	1357(34.56%)	1710(43.55%)	305(7.75%)
	Primary	669(17.02%)	746(19.01%)	991(25.23%)	424(10.80%)
	Secondary	252(6.41%)	93(2.36%)	165(4.21%)	179(4.56%)
	Higher	121(3.08%)	32(0.81)	34(0.87%)	119(3.02%)
Wealth status	Poor	477(12.15%)	1170(29.79%)	1524(38.81%)	124(3.14%)
	Middle	297(7.57%)	465(11.82%)	701(17.86%)	60(1.53%)
	Rich	924(23.53%)	594(15.13%)	675(17.19%)	843(21.47%)
Parity	1	450(11.45%)	436(11.11%)	602 (15.33%)	284(7.22%)
	2-3	638(16.25%)	700(17.83%)	884(22.51%)	454(11.56%)
	4+	611(15.56%)	1092(27.81%)	1414(36.01%)	289(7.36%)
	First child	427(10.85%)	399(10.16%)	545(13.87%)	281(7.14%)

Birth order	2-3 children	615(15.66%)	661(16.84%)	832(21.19%)	444(11.31%)
	4-5 children	354(9.01%)	495(12.61%)	712(18.14%)	137(3.49%)
	6+ children	303(7.72%)	673(17.13%)	811(20.66%)	165(4.20%)
Residence	Urban	610(15.52)	417(10.62)	-	-
	Rural	1089(27.73)	1811(46.13)	-	-
Region	Pastoralist	76(1.95%)	259(6.59%)	229(5.84%)	107 (2.71%)
	Urban	118(3.01%)	30(0.75%)	11(0.27%)	137 (3.49%)
	Agrarian	1504(38.30%)	1940(49.40%)	2660(67.76%)	783(19.94%)

*- Catholic and traditional religion follower

Rural-Urban Disparities in full antenatal care utilization among reproductive age women in Ethiopia

The prevalence of full antenatal care services uptake was 43.25% (95% CI: 41.7%, 44.8%). The difference in the prevalence of full antenatal care service uptake between rural and urban areas was ~~high~~ (rural prevalence was 27.73%, while in urban areas it was 15.52%).

Decomposition Result

The detail decomposition result showed that there is a significant disparity in full antenatal care between rural urban residences (-0.218, $p < 0.001$). The difference in full antenatal care between rural and urban residencies was explained by a component of compositional differences (covariate distribution), which accounted for 76.84% and 23.16% was supplied by a component of difference in behaviour (the effect of covariate differences).

Difference in characteristics (covariate distribution)

The difference in characteristics (endowments) accounted for 76.84% of the observed residence differences in full antenatal care uptake, with high intercept differences (-0.17, $p<0.001$).

The majority of the gap in full antenatal care utilization was explained by wealth status difference between rural and urban women; both poor (30.28%) and middle wealth status (8.92%) distribution contributed for widening of the gap. Shifting rural pastoralist (-3.28%) distribution to urban levels contributed for narrowing of the gap. After controlling for other factors, the distribution of women education with primary (3.76%), secondary (11.84%), and higher (15.27%) education level would be expected to widening rural-urban full antenatal care utilization disparity. That is, if urban women distribution of primary, secondary and higher education shifted to rural levels the rural-urban disparity would increase by 3.76%, 11.84%, and 15.27% respectively. The distribution of religion of women, both Orthodox Christian (0.80%) and other (catholic& traditional) (2.37%) religion followers contributed for widening of the gap. That is, if urban women distribution of orthodox christian and catholic & traditional religion follower shifted to rural levels the rural-urban disparity would increase by 0.80% and 2.37% respectively.

Difference due to coefficients (the effect of covariate differences)

Differences in effects (difference due to coefficients) account for 23.16% of the observed rural- urban disparity in full antenatal care utilization.

The effect of catholic and traditional (1.13%) religion followers contribute for widening the gap of full antenatal care utilization between rural and urban women. The effect of having one living children (one parity) (69.29%) contributes for widening the gap of full antenatal services utilization between rural and urban women. Whereas, the effect of birth order of first

child (-68.68%) contributes for narrowing the gap of full antenatal care utilization between rural and urban women if the urban women birth order shifts to rural level. (Table 2)

Table 2: Detailed decomposition of full antenatal care utilization by place of residence among reproductive age women using 2019 MEDHS, 2023

**Decomposition**	**Coefficient with 95% CI**	**Percent**	**P-value**			
Raw Difference	-0.21807 (-0.27397, -0.16217)	100	0.000			
Explained(E)	-0.16756 (-0.20616, -0.12896)	76.84	0.000			
Unexplained(C)	-0.05051 (-0.11642, 0.01541)	23.16	0.133			
	**Difference due to characteristics (E)**			**Difference due to coefficients (C)**		
	**Coefficient**	**Percent**	**P –value**	**Coefficient**	**Percent**	**P -value**
**Age in years**						
15-24	0.00219	-1.00	0.053	-0.00838	3.84	0.750
25-34	-0.00023	0.10	0.858	-0.00913	4.19	0.814
35-49	1			1		
**Marital status**						
Currently Married	0.00342	-1.57	0.061	-0.12179	55.85	0.147
Currently not married	1			1		
**Religion**						
Orthodox Christian	-0.00175	0.80	0.039	0.01600	-7.34	0.532
Muslim	1			1		
Protestant	0.00240	-1.10	0.231	0.03251	-14.91	0.153
Other*	-0.00517	2.37	0.010	-0.00247	1.13	0.010
**Women education**						
No education	1			1		

Primary	-0.00819	3.76	<0.001	0.01237	-5.67	0.643
Secondary	-0.02583	11.84	<0.001	-0.01870	8.57	0.240
Higher	-0.03329	15.27	0.013	0.00321	-1.47	0.847
**Region**						
Pastoralist	0.00716	-3.28	<0.001	-0.00240	1.10	0.754
Urban	1			1		
Agrarian	-0.01262	5.79	0.094	0.03709	-17.01	0.402
**Wealth status**						
Poor	-0.06586	30.28	<0.001	0.00450	-2.06	0.674
Middle	-0.01945	8.92	0.004	0.00534	-2.45	0.436
Rich	1			1		
**Parity**						
1	0.00124	-0.57	0.866	-0.015110	69.29	0.036
2-3	1			1		
4+	-0.00334	1.53	0.813	-0.07675	35.19	0.174
**Birth order**						
First child	-0.00393	1.80	0.674	0.14978	-68.68	0.038
2-3 children	1			1		
4-5 children	0.00215	-0.98	0.783	0.03368	-15.45	0.184
6+ children	-0.00646	2.96	0.496	0.03261	-14.95	0.344

*- Catholic and traditional religion follower

Discussion

This study showed that the prevalence of full antenatal care utilization was 43.25% (95% CI: 41.7%, 44.8%). The difference in the prevalence of full antenatal care utilization between rural and urban areas was high. More than three-fourths of the observed rural-urban full antenatal care utilization disparities among women could be attributed to differences in composition. This implies that the rural-urban gap in full antenatal care would be reduced more by changes in characteristics (endowment) than change in behavior (coefficient). This finding is supported by previous studies conducted in Ethiopia(14, 24), Sub‑Saharan Africa(8), and Vietnam(25), found that a significant difference in full antenatal care utilization between rural and urban resident. This might be because the women having rich wealth status, educated mothers, living in urban region and Muslim in religion have a better chance of utilizing optimum antenatal care service.

The findings of this study showed that women education was an important predictor for widening the rural-urban disparities in full antenatal care utilization. Shifting urban women education level of primary, secondary and higher to rural levels would provide the increase in gaps or equalizing rural women's educational status to that of urban women at the primary, secondary and higher educational levels would decrease the rural-urban disparity in full antenatal care uptake. This finding is supported by results in Ethiopia (12-14, 24), Sub‑Saharan Africa(8) and North India(26). The possible justification of this finding might be that women, who are educated more, tend to use antenatal care, have better understanding of information and have better knowledge about importance of the service. Moreover, educated women are more likely to improve independence, self-confidence and ability to make decisions about their own health and seek out higher quality services and greater ability to use health care inputs that offer better care(13).

This study revealed that shifting urban poor and middle wealth status to rural levels would provide the largest increase in gaps of rural-urban full antenatal care utilization. This result is concurrent with studies conducted in Ethiopia (13, 14, 24), Sub‑ Saharan Africa(8), and North India(26). It is obvious that rich women in urban areas have easy access to information regarding maternal and child health through different media, which helps them to have ANC follow up. However, rural women of poor and middle wealth status might be incapable to cover transportation and other related expenses to access the health facility, even though the ANC service is free from charge so due to such reasons they might not utilize the service.

Shifting urban distribution of both orthodox christian and other (catholic& traditional) religion followers to rural levels would be expected to increase the gap of full antenatal care uptake between rural and urban women. The possible explanation might be rural women of orthodox christian followers and women having traditional beliefs may not utilize service because those women might be conservative regarding to such service utilization. On the other hand women who live in urban may not have negative attitude regarding maternal health service utilization. Moreover, urban women can access different media which helps to understand the relevance of the service and this enables to utilize ANC.

~~Our~~ finding showed that shifting rural pastoralist distribution to urban levels would significantly contributed for narrowing of the gap. This finding is similar to that of previous study conducted in Ethiopia (15, 16, 24). This could be because women residing in urban regions are capable of getting better information regarding ANC service and have better infrastructure (physical access to health facility) than rural women.

The finding of this study showed that the effect of having one living children (being Para one) contributes for widening the gap of full antenatal care utilization between rural and

urban women. This implies rural para one women may have limited information about this service as compared to their counterparts. However, urban women have better health seeking behavior due to access to information and infrastructures. So the effect of shifting urban Para one women to that of rural level would be expected to widening the gap of full antenatal care utilization between rural and urban women.

Limitations and Strength

One limitation is the cross-sectional nature of the study design limits the ability to establish temporal relationship. Second, both the dependent and independent variables were self-reported and are likely to have a risk of recall bias. Moreover, this analysis considers a few variables recorded in Mini EDHS survey and there are variables not collected as that of main EDHS survey like media exposure, husband education, occupation, even though that are not the only factors which affect FANC uptake. Despite these limitations, this study is important in that it gives an understanding and quantification of the contributors and magnitude of full antenatal care service uptake inequalities in rural-urban settings of Ethiopia. And this enables to give more attention for that of disadvantageous group while planning and implementing strategic interventions targeting to that of drivers of these disparities.

Conclusion and recommendations

There was a significant rural-urban disparity in full antenatal care service uptake among reproductive age women in Ethiopia. A large portion of the rural-urban disparity in full antenatal care service uptake was explained by an endowment (differences in characteristics). Women's educational status, wealth status, region, parity, and religion of women were the determinants of the rural-urban disparity in full antenatal care service uptake. To narrow the rural-urban full antenatal care service uptake disparity, emphasis should be given to both resource distribution targeting to rural households and the appropriate utilization of available

interventions, including improvement of maternal education and creating a plat form to access information about the service and its relevance.

List of abbreviations

CSA: Central Statistical Agency, EA: Enumeration Area, FANC: Full Antenatal Care, ICF: Inner City Fund, IFA: Iron Folic Acid, MEDHS: Mini Ethiopian Demographic Health Survey, PHC: Primary Health Care, STATA, WHO: World Health Organization

Declarations

Ethical approval and consent to participate

The study used publicly available data from the 2019 Ethiopian Mini Demographic and Health Survey. This survey was approved by Inner City Fund (ICF) international as well as the EPHI Institutional Review Board to ensure that a protocol is in compliance with the U.S Department of Health and Human Services regulations for the protection of human subjects. Written informed consent was obtained from study participants and/or their parents or legal guardian(s). Participants were informed that participation was on a voluntary basis. The survey data were received from the DHS International Program upon submission of a proposal. Confidentiality was maintained after data access was authorized by DHS. All methods were performed in accordance with the Declaration of Helsinki and Ethiopian research guideline.

Consent for publication

NA

Availability of data

The datasets analysed in this study are available from the corresponding author upon reasonable request.

Acknowledgement

We would like to acknowledge IPUMS DHS for providing the requested dataset.

References

1. Organization WH. WHO recommendations on antenatal care for a positive pregnancy experience: executive summary: Geneva: World Health Organization; . 2016.
2. Tunçalp Ö, Were WM, MacLennan C, Oladapo OT, Gülmezoglu AM, Bahl R, et al. Quality of care for pregnant women and newborns-the WHO vision. BJOG : an international journal of obstetrics and gynaecology. 2015;122(8):1045-9.
3. Alkema L, Chou D, Hogan D, Zhang S, Moller A-B, Gemmill A, et al. Global, regional, and national levels and trends in maternal mortality between 1990 and 2015, with scenario-based projections to 2030: a systematic analysis by the UN Maternal Mortality Estimation Inter-Agency Group. The lancet. 2016;387(10017):462-74.
4. Organization WH. TRENDS IN MATERNAL MORTALITY 2000 to 2017 Estimates by WHO, UNICEF, UNFPA, World Bank Group and the United Nations Population Division. 2017.
5. Carroli G, Rooney C, Villar J. How effective is antenatal care in preventing maternal mortality and serious morbidity? An overview of the evidence. Paediatric and perinatal Epidemiology. 2001;15:1-42.
6. Vogel JP, Souza JP, Gülmezoglu AM, Mori R, Lumbiganon P, Qureshi Z, et al. Use of antenatal corticosteroids and tocolytic drugs in preterm births in 29 countries: an analysis of the WHO Multicountry Survey on Maternal and Newborn Health. The Lancet. 2014;384(9957):1869-77.
7. Haftu A, Hagos H, Mehari M-A, G/her B. Pregnant women adherence level to antenatal care visit and its effect on perinatal outcome among mothers in Tigray public health institutions, 2017: cohort study. BMC research notes. 2018;11:1-6.
8. Samuel O, Zewotir T, North D. Decomposing the urban–rural inequalities in the utilisation of maternal health care services: evidence from 27 selected countries in Sub-Saharan Africa. Reproductive Health. 2021;18(1):1-12.
9. ICF. CSACEa. Ethiopia Demographic and Health Survey

2016. Addis Ababa, Ethiopia, and Rockville, Maryland,; 2016. https://dhsprogram.com/pubs/pdf/

FR328/FR328.pdf.; 2016.

10. Dewau R, Muche A, Fentaw Z, Yalew M, Bitew G, Amsalu ET, et al. Time to initiation of antenatal care and its predictors among pregnant women in Ethiopia: Cox-gamma shared frailty model. Plos one. 2021;16(2):e0246349.

11. Ewunetie AA, Munea AM, Meselu BT, Simeneh MM, Meteku BT. DELAY on first antenatal care visit and its associated factors among pregnant women in public health facilities of Debre Markos town, North West Ethiopia. BMC pregnancy and childbirth. 2018;18(1):1-8.

12. Gebresilassie B, Belete T, Tilahun W, Berhane B, Gebresilassie S. Timing of first antenatal care attendance and associated factors among pregnant women in public health institutions of Axum town, Tigray, Ethiopia, 2017: a mixed design study. BMC pregnancy and childbirth. 2019;19:1-11.

13. Tsegaye B, Ayalew M. Prevalence and factors associated with antenatal care utilization in Ethiopia: an evidence from demographic health survey 2016. BMC Pregnancy and Childbirth. 2020;20(1):1-9.

14. Tsegaye S, Yibeltal K, Zelealem H, Worku W, Demissie M, Worku A, et al. The unfinished agenda and inequality gaps in antenatal care coverage in Ethiopia. BMC pregnancy and childbirth. 2022;22(1):82.

15. Yehualashet DE, Seboka BT, Tesfa GA, Mamo TT, Seid E. Determinants of optimal antenatal care visit among pregnant women in Ethiopia: a multilevel analysis of Ethiopian mini demographic health survey 2019 data. Reproductive Health. 2022;19(1):1-8.

16. Tegegne TK, Chojenta C, Getachew T, Smith R, Loxton D. Antenatal care use in Ethiopia: a spatial and multilevel analysis. BMC pregnancy and childbirth. 2019;19:1-16.

17. Agency CS. Ethiopia Mini Demographic Health Survey. 2019.

18. Wado YD, Gurmu E, Tilahun T, Bangha M. Contextual influences on the choice of long-acting reversible and permanent contraception in Ethiopia: a multilevel analysis. PloS one. 2019;14(1):e0209602.

19. Ethiopia MoH. National Guideline for Family Planning Services in Ethiopia. 2020.

20. Oaxaca R. Male-female wage differentials in urban labor markets. International economic review. 1973:693-709.

21. Powers DA, Yoshioka H, Yun M-S. mvdcmp: Multivariate decomposition for nonlinear response models. The Stata Journal. 2011;11(4):556-76.
22. Yun M-S. Decomposing differences in the first moment. Economics letters. 2004;82(2):275-80.
23. Yun M-S. Identification problem and detailed Oaxaca decomposition: a general solution and inference. Journal of economic and social measurement. 2008;33(1):27-38.
24. Yesuf EA, Calderon-Margalit R. Disparities in the use of antenatal care service in Ethiopia over a period of fifteen years. BMC pregnancy and childbirth. 2013;13(1):1-10.
25. Tran TK, Nguyen CT, Nguyen HD, Eriksson B, Bondjers G, Gottvall K, et al. Urban-rural disparities in antenatal care utilization: a study of two cohorts of pregnant women in Vietnam. BMC health services research. 2011;11(1):1-9.
26. Pallikadavath S, Foss M, Stones RW. Antenatal care: provision and inequality in rural north India. Social science & medicine. 2004;59(6):1147-58.
