# Supplementary material for: Rural-urban disparities in full antenatal care utilization among women in Ethiopia: A further analysis of Mini-EDHS, 2019
Source: PLoS One. 2024 Nov 18;19(11):e0310901. doi: 10.1371/journal.pone.0310901 (PMC11573122; doi:10.1371/journal.pone.0310901)
Supplement: Attachment — Submitted filename: Response to Reviwers.docx [file pone.0310901.s003.docx]

**Responses to Editor and Reviewers**
Dear to Editor and Reviewers;

We are very thankful for your invaluable comments given. Here are the authors’ responses to the comments.
We have attached the revised manuscript based on your comments.

**Editor comments:**

**Comment 1:** Please ensure that your manuscript meets plos one's style requirements, including those for file naming.

**Response 1:** We have taken your comments and have been revised according to the journal requirement.

**Comment 2:** Please ensure that you have an ORCID ID and that it is validated in editorial manager.

**Response 2:** I have an ORCID ID and is validated in editorial manager

**Comment 3:** We noticed you have some minor occurrence of overlapping text with the following previous publication(s), which needs to be addressed

**Response 3:** We have checked and rephrased some texts which seems having overlapping.

**Comment 4:** We note that you have indicated that data from this study are available upon request.

**Response 4:** We have attached the dataset that were analysed in this study as supplementary file in our revision.

**Comment 5:** please ensure that your abstract adheres to our instructions for Author's formatting guidelines.

**Response 5:** Thank you and your comment take into account.

**Comment 6:** Kindly include the requested supplementary file.

**Response 6:** We have attached it.

**Comment 7:** Ensure consistency in the description of your study design (is this a community based cross-sectional study?)

**Response 7:** Thank you and we have corrected like this a nationwide representative sample of the Mini Demographic and Health Survey (DHS) of Ethiopia.

**Comment 8:** Offer clear operational definitions for “urban” and “rural”.

**Response 8:** We have used place of residency as underlying stratifier variables which is predefined during the survey and in our case we were coded rural as “1” and urban as “0”.

**Comment 9:** Thoroughly depict the study area, period, design, data sources, study population, sample size determination method, sampling technique, inclusion and exclusion criteria, and other pertinent details.

**Response 9:** Thank you and we have revised all sections of methods in our manuscript.

**Reviewer1
Reviewer #1: Comment:** What is the new and novel finding of this research works?

**Response:** Thank you and in Ethiopia research findings showed a difference in full ANC utilization between rural and urban residents. But it was not rigorously explained using the decomposition method yet and the factors were not well established for this difference to design reasonable interventions. Therefore, this study was designed to decompose the factor that significantly contributes for these rural-urban disparities in full antenatal care utilization.

**Reviewer# 2: Comments:**

**Comment 1:** Depth of the issue e.g. MOH ANC new guideline was not cited

**Response 1:** Thank you for reminding, but we were citing the recent WHO guideline (WHO recommendations on antenatal care for a positive pregnancy experience) which recommends eight visit, but in this study we have taken a woman having at least four ANC visit as “yes” and less than 4 ANC visits as “no”. Because our study were based on secondary data. **Comment 2:** The methods is not clear and the selection of variables need strong conviction

**Response 2:** We have taken your comment and review our method section and since we have used secondary data the selection of variables depend on the data at hand meaning that we have included those variables included in this survey, though this Mini EDHS did not include all variables as that of Main EDHS. Because as we all understand that this survey was conducted to observe the progress of health and health related conditions focused on some indicators and did not include all variables as that of Main EDHS and we include this as limitation of the study. Please see the manuscript.

**Comment 3:** The title, the objectives and the contents are somewhat inconsistent

**Response 3:** Thank you and we have addressed your concerns.

**Comment 4:** The consistency between the title and the contents and objective and role of authors should clearly stipulated

**Response 4:** We have taken all your comments and corrected them accordingly. But as per our understanding the role or contribution of authors have been clearly added during submission.

**Comment 5:** You did not cite the DHS in some parts and even the citation is wrong. E.g. see the reference section. E.g. the sampling procedure and definitions are already stated in EDHS itself.

**Response 5:** Thank you, but references were inserted for sampling procedure and operational definitions.

**Comment 6:** In general, all sections need meticulous explanation and justification.

**Response 6:** Thank you for your comments and we see our manuscript thoroughly and revised accordingly.

**Comment 7:** Try to change the majority of the article

**Response 7:** Thank you and we have revised the whole section of the manuscript.
