# Supplementary material for: Rural-urban disparities in full antenatal care utilization among women in Ethiopia: A further analysis of Mini-EDHS, 2019
Source: PLoS One. 2024 Nov 18;19(11):e0310901. doi: 10.1371/journal.pone.0310901 (PMC11573122; doi:10.1371/journal.pone.0310901)
Supplement: Attachment — Submitted filename: Question to be answered, deco.docx [file pone.0310901.s005.docx]

Question to be answered

1. What basic observational disparities the authors observed to do this decomposistion analysis inEthiopia?
2. Why not the authors accounted the effect of endowment and coeffient interaction explanation on the gaps of ANC utilization?
3. There are need of presentation of some findings further by tables/graphs, but missed. For example, line numbers 192 and 205
4. What will be the authors scientific recommendation for the covariate religion explanation for ANC urban-rural disparity? I recommend you to omit.
5. Why the authors lack similar findings of religious difference? Can you declare that it is a novel finding?

The overall comments and some questions are highlighted in the manuscript.
