# Supplementary material for: Rural-urban disparities in full antenatal care utilization among women in Ethiopia: A further analysis of Mini-EDHS, 2019
Source: PLoS One. 2024 Nov 18;19(11):e0310901. doi: 10.1371/journal.pone.0310901 (PMC11573122; doi:10.1371/journal.pone.0310901)
Supplement: Attachment — Submitted filename: Second Response to Reviwers .docx [file pone.0310901.s006.docx]

**Responses to Editor and Reviewers**
Dear to Editor and Reviewers;

We are very thankful for your constructive comments and suggestions. Here under are

authors’ responses to the comments raised during second revision.

We have attached the revised manuscript based on your comments.

**Editor comment:**

**Comment:** The manuscript you submit is good. However, it can improve if you take into consideration the suggestions of the reviewers.

Regarding to the supporting information (data set) we upload the data set, but you can access the full dataset in STATA file format from MEASURE DHS website: [**https://dhsprogram.com**](https://dhsprogram.com)

**Response 1:** Thank you and we have considered all the comments and suggestions raised by all reviewers.

**Reviewer 2:** Thank you for recognizing our previous review response effort. Your encouragement to conduct research with the team is greatly appreciated, as your contribution significantly enhances the soundness of our work for the scientific community. **Reviewer #2: Comments:**

**Comment 1:** On the title: Needs reframing and make it absorbing to the reader. For instance, If I were you, I may rewrite the title as “Rural-urban disparities in full antenatal care utilization among women in Ethiopia: A further Analysis of mini-EDHS, 2019”

**Response 1:** Thank you for your constructive comment and we have re-write the title.

**Comment 2:** On the abstract section; background is incomplete, it lacks clarity and used inappropriate words E.g., ‘to see’, and the presentation of the result is also incomplete E.g., lacks confidence interval.

**Response 2:** Thank you for your comment and we have corrected accordingly, but regarding to result part the purpose of this study was to observe the difference in FANC utilization that is explained by residences difference in the level of the determinants (covariate effects), and by differences in the effect of the covariates on the outcome (coefficient effects). So in result the overall prevalence of FANC utilization was presented with its 95% CI and we include the disparity in full antenatal care utilization of rural urban resident women with its 95% CI considering your comment. Moreover to this, the characteristics and effects components do not provide information about the precision of the contributions to group differences.

**Comment 3:** **On the methods section;**

**3.1:** It is known that urban dwelling women have higher full ANC utilization when compared with rural dwellers due to the gains in the urban dwelling. Hence, what new findings does this study report?

**Response:** Thank you for your comment and as you stated that urban resident women have higher full ANC utilization when compared with rural resident due to the opportunities in the urban and in Ethiopia previous research findings showed a difference in full ANC utilization between rural and urban residents. But it was not rigorously explained using the decomposition method yet and the factors were not well established for this difference to design reasonable interventions. Therefore, this study was designed to decompose the factor that significantly contributes for these rural-urban disparities in full antenatal care utilization. Meaning that this study result showed that what contributes for the gap (demographic composition of the variable or behavioural change).

**3.2:** Why was the specific type of analysis then employed?

**Response:** **Multivariate decomposition analysis was employed and it** is a statistical technique used to understand the differences in an outcome variable across groups by decomposing these differences into contributions between groups into explained (due to predictors) and unexplained (due to differences in coefficients) components. Therefore this study enables to identify the source of variation in full antenatal care utilization between the rural and urban areas and to design appropriate intervention strategies and programs which minimizes the gap between rural and urban residence women.

**3.3:** What was the reason behind the selection of those women aged 15 to 49 years (3979)?

**Response:** The first thing is that women aged 15 to 49 years are target to study ante natal care utilization. Moreover, to this we were using secondary data(mini EDHS 2019) and the data were collected among women aged 15-49 who had a live birth in the 5 years preceding the survey with four or more ANC visits for the most recent live birth.

**3.4:** The number of sampled women before your analysis in the actual EDHS, and yours (3,979)?

**Response:** The target women interviewed were 3979 in the actual EDHS, but we use sample weighting during analysis to consider clustering effect of sampling to produce proper representation.

**Comment 3: On the result and the consequent sections**

**3.1:** The rural dwellers have higher full ANC utilization that the urban one. Access, sampling?

**Response:** Thank you for your comment and the reason is sampling

**3.2:** Try to address the similarity and the difference between the regions and ‘cultural difference’?

**Response:** Thank you and we have stated our finding related to region clearly.

**3.3:** It lacks proper description of the findings E.g., Use of figure.

**Response:** Thank you for your comment, but we consider this issue while writing our report and we can use for example bar graph for some variables finding for explained or unexplained components with percentage, but this way of presenting the result was not comprehensive because without the estimate (coefficient) and p- value that our interpretation bases on it did not be self-explanatory by itself meaning that there were a need of tabular presentation also and this is redundancy; that is why preferring tables and text to present the result.

**3.4:** Strengthen the discussion section.

**Response:** We have taken your comment.

**3.5:** The conclusion and the recommendation should be in line with the findings of the study. Likewise, specific recommendation should be conveyed.

**Response:** Thank you and we have addressed your concerns.

**3.6:** The manuscript didn’t declare ‘conflict of interest’

**Response:** Thank you for reminding and we will include it.

**Reviewer #3: Comments:**

**Comment 1:** First of all, I would like to thank the editor for inviting me to review this manuscript. Then, the authors for coming with an important topic.

**Response 1:** We are very grateful for your constructive comments and suggestions.

**Comment 2: Abstract**

The document needs intensive revision for grammatical issues and sentence organization including use of tenses and conjugations in the appropriate place, and punctuations. Suggest to modify the first sentence. To make it sound, don’t start with even though.

**Response 2:** Thank you for your constructive comment and we have corrected accordingly.

**Comment 3: Methods**

**3.1:** Who did cross-sectional study? It is not your data, please refer the source of the data then state how they capture it. Otherwise, it seems you have actively participated in the data collection process.

**Response:** The survey was conducted by the Ethiopian Public Health Institute (EPHI), in partnership with the Central Statistical Agency (CSA) and the Federal Ministry of Health (FMOH). And we cited the source of the data and detail about the process how they conduct the study was written explicitly in the method section.

**3.2:** Suggest replacing “prevalence” by other terms for explaining full antenatal utilization.

**Response:** Thank you. Since a community-based cross-sectional study was conducted, we believe it is appropriate to use the term "prevalence.

**3.3:** Report CI for rural and urban ANC utilization.

**Response:** We have taken your comment.

**3.4:** Rural ANC utilization was higher than urban. Your explanations have not supported your findings. ” So to narrow this gap, emphasis should be given to both resource distribution targeting to rural households, improvement of maternal education and creating a plat form to access information about the service and its relevance”.

**Response:** The rural residences have higher full ANC utilization than the urban one because of sampling, but we apply this decomposition method to see the rural-urban disparities in full antenatal care utilization explained by residence difference into components of endowments and coefficients so our explanation bases about which component highly contribute for this gap and the possible ways to narrow this gap.

**3.5:** Please provide evidence based justification and recommend based on your findings.

**Response:** Thank you and we have addressed your concerns.

**Comment 4: Introduction**

**4.1:** Line 52 and 53: suggest modifying the sentence

**Response:** We have taken your comment

**4.2:** Line 54: use updated reference

**Response:** Thank you for reminding, but this reference is a projection from 2015 to 2030 which is updated literature.

**4.3:** Elaborate the introduction starting from extent of poor ANC utilization, its effect on maternal and new-born health, factors affecting ANC utilization and the observed disparities by country, region, and residence. Consequently, explain the interventions posed before for improving ANC utilization and gap of previous studies for showing disparities. Clearly set the gap. Keep flow of the sentences.

**Response:** We tried to address your concerns.

**4.4:** What is the relevance of studying urban and rural disparities? Reference 22, 14, and 15 also studied disparities in ANC utilization in Ethiopia. What values you added?

**Response:** A Study cited as ref. 22 was time trend decomposition analysis, ref. 15 is a multilevel analysis of Ethiopian mini demographic data to identify the individual and community level factors of optimum ANC and ref. 14 was the same scenario as this one and including from 2000-2019, but measured focused ANC. However, in 2016 WHO revised the recommended minimum number of ANC visits from four to eight contacts. Therefore this study was based on current recommendation and also time value take into account for such like behavioural conditions.

**4.5:** Line 75: “Because though” please revise the document for English grammar.

**Response:** we have corrected it.

**Comment 5: Methods and Materials**

**5.1:** Line 92: better to report study population instead of sampled population.

If a mother provides two or three live births within five years, what measures are taken during sampling? Which ANC was taken? If there are disparities in ANC utilization for different pregnancies within a mother, in which category you classified this particular participant? This all needs to be clarified.

**Response:** We have taken the recent live birth within five years preceding the survey and for a mother having a difference in ANC utilization the category was based on the most recent one because ANC utilization is affected by health seeking behaviour and due to the implementation of different policies like MDGs, HSTP and SGDs we expected that this all would contribute to change maternal health service uptake.

**Comment 6: Results**

**6.1:** Suggest providing clear interpretation for your decomposition results. “That is, if urban women distribution of primary, secondary and higher education shifted to rural levels the rural-urban disparity would increase by 3.76%, 11.84%, and 15.27% respectively”

**Response:** Thank you for your constructive comment and we corrected as “That is, if urban women distribution of primary, secondary and higher education shifted to rural levels the rural-urban disparity would narrowed (decreased) by 3.76%, 11.84%, and 15.27% respectively”. And also we revised our result and discussion section as a whole considering some editorial and interpretation problems.

**6.2:** Suggest describing about difference due to characteristics and difference due to coefficients in methods section.

## Response: It was clearly written in data processing and analysis part.

## Comment 7: Discussion

**7.1:** Inconsistent result is reported in result and discussion section. You reported “The disparity of full antenatal care utilization between rural and urban areas was high (rural prevalence was 27.73%, while in urban areas it was 15.52%)” , whereas in result section you reported “The result of this study showed that full antenatal care utilization among women in urban areas of Ethiopia was better than that of rural residents” in discussion.

**Response:** Thank you; points written as “urban areas of Ethiopia were better than that of rural residents” is editorial problem so we made a correction on the revised manuscript.

**7.2:** Did you consider changes in characteristics (endowment) and change in behaviour (coefficient) as a variable?

**Response:** We considered endowment as variable and coefficient as the effect of endowment (variable).

**7.3:** What is your base for discussing “changes in characteristics (endowment) than change in behaviour (coefficient)”?

**Response:** We base on three parameters (coefficient (positive or negative), p-value (significant or not) and percentage) to discussion our findings.

**7.4:** Line 227 to 229: suggest providing evidence based justification for the particular finding.

**Response:** We revised it.

**7.5:** How you relate Muslim religion and ANC utilization. Here, you have to provide justification for the disparities occurred between urban and rural areas.

**Response:** Even though we were using secondary data that enumeration area selected might affect the result and such characteristics is difficult for practically applicable recommendation, we share your concern and revised accordingly.

**7.6:** Add practical implication for pertinent findings.

**Response:** We have corrected it.

**7.7:** Your Interpretations are not still clear in discussion. Please provide clear and simple interpretations.

**Response:** We revised our result and discussion section as a whole considering some editorial and interpretation problems.

**7.8:** Line 242 to 244. “This result is in line with studies conducted in Ethiopia (13, 14, 22),” if ample evidences are available in Ethiopia, what you have added????

**Response:** In Ethiopia previous research findings showed a difference in full ANC utilization between rural and urban residents including determinants. But it was not rigorously explained using the decomposition method yet and the factors were not well established for this difference to design reasonable interventions. Therefore, this study was designed to decompose the factor that significantly contributes for these rural-urban disparities in full antenatal care utilization. Meaning that this study result showed that what contributes for the gap (demographic composition of the variable or behavioural change).

**7.9:** You have used almost all references in your discussion from Ethiopia.

**Response:** One thing we have used different references and while writing discussion it is better to discuss with previous studies with comparable scenario.

**7.10:** Line 256: add reference for your justification.

**Response:** We consider your comment.

**Reviewer #4:** **Comments**

**Comment 1:** What basic observational disparities the authors observed to do this decomposition analysis in Ethiopia?

**Response 1:** It is known that urban residence women have higher full ANC utilization when compared with rural one due to the gains in the urban residence women. According to HSTP by the end of 2020 increase proportion of women having at least 4 visits of ANC from 68% to 95% was the national target and SDG 3.1 sets a specific target of MMR reduction below 70 by 2030 in the world, but our study showed magnitude of full antenatal care utilization as 43.25% is progressing, but still it is lagging below the country’s target. Moreover to this, previous researches showed that place of residence has a significant association to ANC (urban and rural women utilize the services differently). Considering this we apply this method to decompose the factors that significantly contributes for these rural-urban disparities in full antenatal care utilization by using residence as equity stratifier variable to show whether due to demographic composition of the variable or behavioural change.

**Comment 2:** Why not the authors accounted the effect of endowment and coefficient interaction explanation on the gaps of ANC utilization?

**Response 2:** The available data might not have permitted a detailed analysis of endowment and coefficient interactions, as the MEDHS included only a limited set variables. Consequently, we aimed to demonstrate the direct effects of endowment and coefficient on ANC utilization, making the findings more directly actionable for policy recommendations and interventions.

**Comment 3:** There are needs of presentation of some findings further by tables/graphs, but missed. For example, line numbers 192 and 205.

**Response 3:** Thank you for your comment, and this issue was raised by reviewer **#,** but we consider this issue while writing our report and we can use for example bar graph for some variables finding for explained or unexplained components with percentage, but this way of presenting the result was not comprehensive because without the estimate (coefficient) and p- value that our interpretation bases on it did not be self-explanatory by itself meaning that there were a need of tabular presentation also and this is redundancy; that is why preferring tables and text to present the result.

**Comment 4:** What will be the author’s scientific recommendation for the covariate religion explanation for ANC urban-rural disparity? I recommend you to omit.

**Response 4:** We have taken your comment.

**Comment 5:** Why the authors lack similar findings of religious difference? Can you declare that it is a novel finding?

**Response 5:** Thank you for your comment, since we used mini EDHS data sampling has its own effect like enumeration areas selected may affect such demographic compositions. So due to this reasons not novel finding and we tried to revise it accordingly in the result and discussion.

**Comments on attached document**

We are very thankful for your constructive comments and suggestions highlighted in the attached manuscript. So we tried to address all the issues raised and you can appreciate on the revised document. In addition to revising the main document here are some responses for the comments raised.

**Responses:**

**1, Introduction part:** We thank you for your valuable comments and we revised accordingly.

**2, Operational definition:** We have taken your comment.

**3, Data processing and analysis:** Majority of the comment were addressed in first round revision, but to address you question

3.1 From where? An organization that permit you to do? CSA DHS program

3.2 Survey design has been taken into account while the analysis was done (make clear for readers really what you did): we clearly state what we were doing as sample weighting was done during analysis to consider clustering effect of sampling to ensure proper representation.

3.3, we have inserted citation

4, **Result:** what is the authors basic justification that used Muslim as a reference? We used a reference for this variable. As we generated and extracted the data, this category was considered to be the default reference.

5, **Discussion:** This justification should be supported by evidences. Currently there are no great wealth differences among urban and rural residences regarding of access to healthcare. Plus ANC is a free of charge service, everyone can access it. Even though the physical accessibility of the healthcare facility is ensured, the acceptability and quality of the actual services by the community matters. Beside to this many are unable to cover transportation and other related expenses to access the health facility, even though ANC services are free of charge. Due to such reasons, they might not utilize the service.

6, **Ethics approval:** Ethical consideration should be presented next to data processing and analysis. We have taken your comment

**Reviewer #5:** **Comments**

There are some places that need to be grammatically checked. I noticed that there are some places that are missing spaces. One such instance is between a word and the reference number. There are places that have spaces and some that don't. Another discrepancy I see is that you put commas for numbers greater than or equal to 1,000 (not referencing a year). Please make sure the spacing between the references follow the PLOS submission guideline. **Response:** Thank you very much for your comment and we have revised thoroughly regarding those issues.
